# Vegetation productivity patterns at high northern latitudes: a multi-sensor satellite data assessment

**DOI:** 10.1111/gcb.12647

**Published:** 2014-07-21

**Authors:** Kevin C Guay, Pieter S A Beck, Logan T Berner, Scott J Goetz, Alessandro Baccini, Wolfgang Buermann

**Affiliations:** 1 The Woods Hole Research Center149 Woods Hole Road, Falmouth, MA, 02540-1644, USA; 2 Forest Resources and Climate Unit, Institute for Environment and Sustainability (IES), Joint Research Centre (JRC), European CommissionVia Enrico Fermi 2749, Ispra, VA, 21027, Italy; 3 Department of Forest Ecosystems and Society, Oregon State University321 Richardson Hall, Corvallis, OR, 97331-5752, USA; 4 Institute for Climate and Atmospheric Science, School of Earth and Environment, University of LeedsLeeds, LS2 9JT, UK

**Keywords:** arctic, boreal, climate change, GIMMS, MODIS NBAR, NDVI3g, normalized difference vegetation index, SeaWiFS, SPOT D10

## Abstract

Satellite-derived indices of photosynthetic activity are the primary data source used to study changes in global vegetation productivity over recent decades. Creating coherent, long-term records of vegetation activity from legacy satellite data sets requires addressing many factors that introduce uncertainties into vegetation index time series. We compared long-term changes in vegetation productivity at high northern latitudes (>50°N), estimated as trends in growing season NDVI derived from the most widely used global NDVI data sets. The comparison included the AVHRR-based GIMMS-NDVI version G (GIMMS_g_) series, and its recent successor version 3g (GIMMS_3g_), as well as the shorter NDVI records generated from the more modern sensors, SeaWiFS, SPOT-VGT, and MODIS. The data sets from the latter two sensors were provided in a form that reduces the effects of surface reflectance associated with solar and view angles. Our analysis revealed large geographic areas, totaling 40% of the study area, where all data sets indicated similar changes in vegetation productivity over their common temporal record, as well as areas where data sets showed conflicting patterns. The newer, GIMMS_3g_ data set showed statistically significant (*α *= 0.05) increases in vegetation productivity (greening) in over 15% of the study area, not seen in its predecessor (GIMMS_g_), whereas the reverse was rare (<3%). The latter has implications for earlier reports on changes in vegetation activity based on GIMMS_g_, particularly in Eurasia where greening is especially pronounced in the GIMMS_3g_ data. Our findings highlight both critical uncertainties and areas of confidence in the assessment of ecosystem-response to climate change using satellite-derived indices of photosynthetic activity. Broader efforts are required to evaluate NDVI time series against field measurements of vegetation growth, primary productivity, recruitment, mortality, and other biological processes in order to better understand ecosystem responses to environmental change over large areas.

## Introduction

The primary productivity of vegetation sets the baseline for the terrestrial carbon sink, transferring carbon from the atmosphere to the biosphere, and is therefore critical to understanding the extent and implications of climate change (Goodale *et al*., 2002; Pan *et al*., 2011). Consequently, substantive changes in vegetation productivity have the potential to either moderate or amplify increases in atmospheric CO_2_ levels (Le Quéré *et al*., 2009). Indeed, atmospheric CO_2_ concentrations over the high northern latitudes have increased in seasonal amplitude during the last half-century. Evidently, this is a result of enhanced carbon uptake by temperate and boreal forests, which is strongly controlled by temperature, albeit moisture-limited in some areas (Wilmking & Juday, 2005; Berner *et al*., 2013). As sustained climate warming is likely to generate profound changes in tundra and boreal biomes (Lotsch *et al*., 2005), the question arises whether these changes will cause an increase or decrease in the terrestrial carbon sink (a negative or positive feedback, respectively; Bond-Lamberty *et al*., 2012). This question is gaining in urgency as evidence mounts that environmental change at high northern latitudes can generate significant positive feedbacks to global warming, including considerable carbon efflux from permafrost soils (Schuur *et al*., 2009; Koven, 2013) and reduced surface reflectivity from vegetation expansion or snow cover loss (Chapin *et al*., 2005; Loranty *et al*., 2013; Pearson *et al*., 2013).

Changes in vegetation productivity and phenology at high northern latitudes were among the first detected biosphere responses to recent climate warming (Myneni *et al*., 1997; Parmesan & Yohe, 2003). With arctic temperatures rising at twice the average global rate, boreal and arctic temperature profiles now resemble those observed in the early 1980s at 5° and 4° latitude further south, respectively, and climate models predict that these profiles will shift by an additional 20° latitude over the course of the 21st century (Xu *et al*., 2013). In response, over half of the terrestrial arctic could experience significant changes in vegetation composition by the middle of this century, including a northward migration of the arctic tree line and associated changes in surface albedo (Pearson *et al*., 2013). At the southern extent of the boreal forest, future climates are increasingly likely to exceed drought tolerances of extant tree species, which combined with altered disturbance regimes, will likely cause losses in ecosystem carbon stocks (Koven, 2013), while also changing albedo dynamics associated with deciduous, rather than evergreen, species dominance (Rogers *et al*., 2013). To anticipate such biome-level shifts, it is critical that ongoing changes in vegetation productivity can be accurately monitored at local to global scales.

Vegetation productivity is most easily studied over large areas using remotely sensed spectral vegetation indices, such as the normalized difference vegetation index (NDVI; Tucker, 1979) and the enhanced vegetation index (EVI; Huete *et al*., 2002). NDVI is the normalized ratio of red and near-infrared (NIR) reflectance, which is influenced by the chemical and structural components of leaves (chlorophyll and mesophyll respectively), and is generally considered a good proxy for photosynthetic activity (Sellers, 1987; Myneni *et al*., 1995; Goetz & Prince, 1999). The GIMMS (Global Inventory Modeling and Mapping Studies) NDVI data set, based on the daily data record from NOAA's Advanced Very High Resolution Radiometer (AVHRR), is the most popular NDVI product for long-term vegetation studies owing to its long, continuous record (starting in July 1981), as well as to its availability and improved performance over other AVHRR-based NDVI data sets (Beck *et al*., 2011a). The GIMMS data set presents more than three decades of NDVI data, in bimonthly intervals, and aims to strike a compromise between combining repeated observations to increase reliability and retaining a temporal resolution in the final product that captures phenological dynamics (e.g., Karlsen *et al*., 2007). The GIMMS-NDVI data sets have been widely used to document variations in the timing of growing seasons, as well as vegetation productivity during the growing season, and how both of these have changed since the early 1980s (Zhou *et al*., 2001; Goetz *et al*., 2005). Studies that focused on the high latitude regions and relied on the GIMMS NDVI record, indicate consistent increases in vegetation productivity, particularly in treeless or sparsely forested areas, concurrent with climate warming, while some areas of dense tree cover show decreases in productivity (Bunn & Goetz, 2006; Verbyla, 2008; Beck & Goetz, 2011). Despite the relatively coarse spatial resolution (˜8 km grid cells) of the GIMMS data, studies comparing the GIMMS record with local measurements of vegetation productivity, using tree and shrub rings, have corroborated these patterns (Beck *et al*., 2011b; Berner *et al*., 2011, 2013; Macias-Fauria *et al*., 2012; Bunn *et al*., 2013; Girardin *et al*., 2014), although reconciling these disparate measurements can be challenging owing to scale mismatches.

While providing a long data record, the legacy AVHRR sensors have a number of drawbacks, including the lack of on-board calibration and inconsistent timing of equatorial crossings (Ignatov *et al*., 2004), as well as differences in the spectral specifications of successive AVHRR sensors (Gleason *et al*., 2002; Trishchenko *et al*., 2002). Newer sensors, such as the Moderate Resolution Imaging Spectroradiometer (MODIS), the Sea-viewing Wide Field-of-view Sensor (SeaWiFS), and the Satellite Pour l'Observation de la Terre (SPOT), provide near-daily global coverage, with higher image resolution and quality than the AVHRR series. As a consequence, global mapping of primary productivity and biomass has, in recent years, relied greatly on data sets derived from these newer sensors, and particularly the suite of MODIS data products (Zhao & Running, 2010; Baccini *et al*., 2012). Furthermore, operational algorithms were developed for MODIS and SPOT reflectance data to account for view and illumination angle effects (Duchemin *et al*., 2000; Schaaf *et al*., 2002), which are known to generate spurious changes in NDVI time series (Holben, 1986; Cihlar *et al*., 1994). Long-term monitoring of productivity trends, using products from modern sensors, is compromised by the relatively short data records, and the challenge of merging data sets generated by instruments with disparate specifications (Tucker *et al*., 2005; Van Leeuwen *et al*., 2006). Thus, legacy data records, such as GIMMS, remain indispensable to studying longer term dynamics of the biosphere for the foreseeable future.

Data sets generated with legacy sensors benefit from those based on newer sensors with respect to calibration and validation. Indeed, the GIMMS-NDVI algorithm relies on data from modern sensors to calibrate between different versions of the AVHRR sensor. Recent NDVI-based studies that map trends in high latitude vegetation growth have evaluated GIMMS-based trends using MODIS data (Beck & Goetz, 2011; Barichivich *et al*., 2013; Xu *et al*., 2013), and some studies have focused exclusively on the comparison of seasonal, or long-term signals, in multiple regional (Baldi *et al*., 2008; Fensholt *et al*., 2009) and global (Beck *et al*., 2011a; Fensholt & Proud, 2012) satellite vegetation data sets. Since these studies were completed, a newer version of GIMMS data (GIMMS_3g_) has been developed, designed specifically to improve data quality in high latitude regions.

We examined how five commonly used NDVI data sets describe the long-term vegetation productivity changes in high latitude regions (north of 50°N). We compared GIMMS_3g_ to its predecessor GIMMS_g_, and incorporated higher resolution NDVI data from MODIS, SeaWiFS and SPOT, as well as, for the first time, MODIS and SPOT NDVI records adjusted for the bidirectional reflectance distribution function (BRDF). In particular, we quantified and compared how each of these data sets describe recent changes in vegetation productivity at high northern latitudes, both over the full temporal extent of each data set, and over the period of temporal overlap (2002–2008). In our analysis, we isolated long-term trends from year-to-year variability to assess the level of consistency at both time scales among the various data sets in capturing ongoing changes in vegetation productivity in different northern vegetation types and at different time scales.

## Materials and methods

### Data sets

The GIMMS_g_ data set, first released in 2004 (Tucker *et al*., 2004; Pinzón *et al*., 2005), uses data collected from the AVHRR sensors aboard NOAA-7, 9, 11, 14, 16, and 17 (Figure S1; Pinzón *et al*., 2007) and has since been updated to cover the period 1982–2008. GIMMS_g_ is a ca. 8 km (0.072°) resolution, bimonthly, maximum value composite (MVC), global NDVI product. The GIMMS algorithm uses empirical mode decomposition (EMD) to separate the surface NDVI reflectance from signal interference, which includes solar zenith angle and cloud contamination, but does not directly correct for bidirectional reflectance (Pinzón *et al*., 2005). While the AVHRR instrument's variable equatorial passing time (Pinzón *et al*., 2007) and lack of onboard calibration make it nearly impossible to correct for bidirectional reflectance, *ex post facto* corrections for satellite drift and atmospheric aerosols have been applied to GIMMS. Two versions of the AVHRR sensor, AVHRR/2 and AVHRR/3, were used in the GIMMS_g_ data set. AVHRR/2 is a 5-channel instrument, which was first launched on NOAA-7 in 1981, while AVHRR/3 has 6 channels and was first launched in May 1998 (Figure S1). Since the two instruments have different spectral bandwidths, GIMMS_g_ uses SPOT VGT data to join observations from AVHRR/2 and AVHRR/3. Due to its calibration with SPOT VGT, whose northern spatial limit is 72°N, GIMMS_g_ has a documented discontinuity north of that latitude (Pinzón & Tucker, 2010), resulting in anomalously low NDVI values. The publically available version of the GIMMS_g_ data set has not been updated since 2008 and was recently replaced by a revised data set, GIMMS_3g_. Nevertheless, GIMMS_g_ is included in this data set comparison because of its widespread use in the analysis of global vegetation dynamics over the past decade (e.g., Beck & Goetz, 2011; Fensholt *et al*., 2012; De Jong *et al*., 2013).

Like its predecessor, the third generation of the GIMMS data set (GIMMS_3g_) is a ca. 8 km resolution, 15-day MVC, bimonthly, global NDVI product generated from AVHRR data. This product integrates data from NOAA-17 and -18, to improve the length and quality of the GIMMS-NDVI record, and enhances techniques used in the data processing with respect to high northern latitudes. The discontinuity north of 72°N, present in GIMMS_g_, was addressed in GIMMS_3g_ by using SeaWiFS (along with SPOT VGT) to combine the AVHRR/2 and AVHRR/3 data sets. The GIMMS_3g_ algorithm also has improved snow-melt detection and is calibrated based on data from the shorter, arctic growing season (May–September), rather than the entire year (January–December).

The Moderate Resolution Imaging Spectroradiometers (MODIS), aboard NASA's Earth Observation System (EOS) Terra and Aqua satellites, each image the earth's surface daily at a spatial resolution of 232 m. Satellite mapping, especially when based on optical sensors, is challenging in the boreal regions because of snow, cloud cover and large solar zenith angles. To overcome these issues, we used an algorithm (Baccini *et al*., 2008, 2012) that combines time series observations of MODIS Nadir BRDF-Adjusted Surface Reflectance (NBAR) data (MCD43A4 V5; Schaaf *et al*., 2002) and a BRDF quality product (MCD43A2) to generate a cloud and snow-free data set of reflectance, modeled as if it was collected at nadir view. These data have been corrected for atmospheric effects, as well as solar and view geometry, and have been screened for cloud cover. In addition, we used quality flag information, included with the MODIS NBAR data, to screen for snow-contaminated pixels. To further ensure data quality, we only used NBAR data that were derived from full BRDF model inversions, or ‘magnitude inversions’, based on at least three observations during two, eight-day compositing periods (after Baccini *et al*., 2008, 2012). Based on the data processing, described above, we generated a composite of monthly average reflectance, for the red and NIR bands, during the period 2001–2011, that are cloud free, snow free, filtered for atmospheric aerosols, and adjusted to resemble observations at local solar noon.

The SeaWiFS instrument aboard the OrbView-2 satellite, previously known as SeaStar, imaged the earth daily from 1998 to 2011 at a spatial resolution of 1.1 km. SeaWiFS had on-board, monthly lunar calibration, in addition to *ex post facto* calibration with MODIS field sites. Although not intended for terrestrial studies, the sensor's sun-synchronous orbit and spectral range (660–680 nm and 845–885 nm for the red and NIR bands respectively; Figure S2), make it possible to calculate NDVI (McClain *et al*., 2004). The 4 km NDVI data set has been used in multiple vegetation studies (e.g., Behrenfeld *et al*., 2000; Wang *et al*., 2001), including NDVI data set comparisons (Brown *et al*., 2006). In mid-2008, both the OrbView-2 satellite and the SeaWiFS sensor started experiencing malfunctions and data interruptions that lasted until the end of the mission in February 2011. For that reason, this study excludes observations since 2008 from the SeaWiFS NDVI record.

SPOT VEGETATION (VGT) is an NDVI product derived from the Satellite Pour l'Observation de la Terre (SPOT). SPOT is a polar orbiting, sun-synchronous satellite that images the earth once daily. SPOT VGT S10 is a ten-day MVC NDVI product, with a 1 km spatial resolution, as well as atmospheric and aerosol corrections (Duchemin & Maisongrande, 2002; Duchemin *et al*., 2002). The D10 version of the SPOT data set (Duchemin *et al*., 2000), used in this study, improves upon the S10 version by implementing a BRDF-based normalization. In addition to the atmospheric and aerosol corrections in the SPOT S10 data, clouds are masked in the D10 product. SPOT D10 uses Bi-Directional Compositing (BDC), which differs from the MVC used in SPOT S10, GIMMS and SeaWiFS, in that it averages the last 12 cloud-free data (regardless of acquisition date) and uses the Roujean's BRDF model (Roujean *et al*., 1992) to calculate the BRDF-corrected NDVI. The BDC method is designed to maintain the spatial and temporal integrity of the NDVI data while reducing the ‘noise’ that the MVC method does not address (Duchemin *et al*., 2000). Table 1 in Brown *et al*. (2006) summarizes the spatial and temporal resolution, equatorial crossing and field-of-view for each sensor used in this study.

**Table 1:** Percent of the study area where the GIMMS_g_ and GIMMS_3g_ data sets show GS-NDVI trends in the same direction (i.e., both greening or both browning) or in opposite directions (i.e., one greening and one browning). Includes naturally vegetated land between 50°N and 72°N, excluding croplands

GIMMS_g_	GIMMS_3g_			
	Greening	Sig. Greening	Browning	Sig. Browning
Greening	14.51	11.73	3.79	0.45
Sig. Greening	3.42	8.61	0.41	0.007
Browning	19.50	7.92	12.57	1.94
Sig. Browning	4.60	1.48	5.45	3.60

### Satellite data preprocessing

The GIMMS_g_, GIMMS_3g_, MODIS NBAR (MODIS), SPOT D10 (SPOT), and SeaWiFS NDVI data sets were projected to the Polar Lambert Azimuthal Equal Area projection, and bilinearly resampled to match the GIMMS_3g_ grid. All five NDVI data sets were then spatially mean aggregated to 24 km grid cells, i.e., 3 × 3 GIMMS_3g_ cells, to reduce any georegistration artifacts in the data at their native resolution. Spatially aggregated 24 km grid cells that were calculated using a considerable number of cells (>20%) with missing data (e.g., cells that fall on the border of land and water) were excluded from this analysis. Observations from GIMMS and SPOT (15 and 10 day temporal resolution respectively) were temporally aggregated to monthly maximum value composites to match the monthly temporal resolution of MODIS and SeaWiFS. Monthly products for June, July, and August were then averaged to create annual growing season NDVI (GS-NDVI) maps. Since NDVI scales non-linearly with the individual red and NIR surface reflectance, the corresponding MODIS bands were projected, resampled, and aggregated to match the GIMMS_3g_ footprint before calculating NDVI. GIMMS, SeaWiFS, and SPOT were downloaded as NDVI products, so the transformations (i.e., projection, resampling, and aggregation) were done on the NDVI rather than the individual red and NIR bands.

### Statistical analysis

After pre-whitening the GS-NDVI time series to remove lag-1 correlation (Figure S3; Zhang *et al*., 2000; Wang & Swail, 2001), the temporal trends in the time series were estimated using the Theil–Sen approach (Theil, 1992). Trends were not estimated for pixels that were missing data anywhere in the time series. The Mann–Kendall test was used to assess trends for statistical significance (Mann, 1945), regarding pixels as significant when *P *<* *0.05. Compared to a simple linear regression, the Theil–Sen slope estimator is more robust to outliers, as it estimates the slope of a time series as the median of all slopes between pairs of observations in the time series. We explored using this population of slopes to empirically determine 95% confidence intervals around the slope estimate as an alternative assessment of statistical significance, however, the Mann–Kendall test was far more conservative when applied to the common record. Trend analyses were performed using the tools in the zyp package (Bronaugh & Werner, 2012) in R (R Core Team, 2012).

Over the entire study area, trends in GS-NDVI for each data set were grouped based on their sign (positive or negative), as well as statistical significance (*P *<* *0.05), and then cross tabulated in pairs of data sets over their common temporal record. Trends were first cross-tabulated based on their sign only, and summarized using Cohen's kappa (Cohen, 1960) to evaluate general temporal agreement between pairs of GS-NDVI data sets. Non-significant trends were then assigned to a separate class and kappa recalculated to evaluate whether different data sets consistently mapped significant increases and decreases in GS-NDVI. In addition to evaluating GS-NDVI data sets for their long-term trends, we also evaluated whether they ranked GS-NDVI similarly at interannual time scales. To this effect, we calculated a rank-based correlation coefficient, Kendall's tau, on detrended GS-NDVI for pairs of data sets as well as on a per-pixel basis. The data sets were detrended using residuals from a simple linear regression. Tau reaches one when the two data sets rank years identically based on GS-NDVI, and negative one when they rank them in opposite orders.

## Results

### Comparison of GIMMS_g_ and GIMMS_3g_

**Fig 1:**
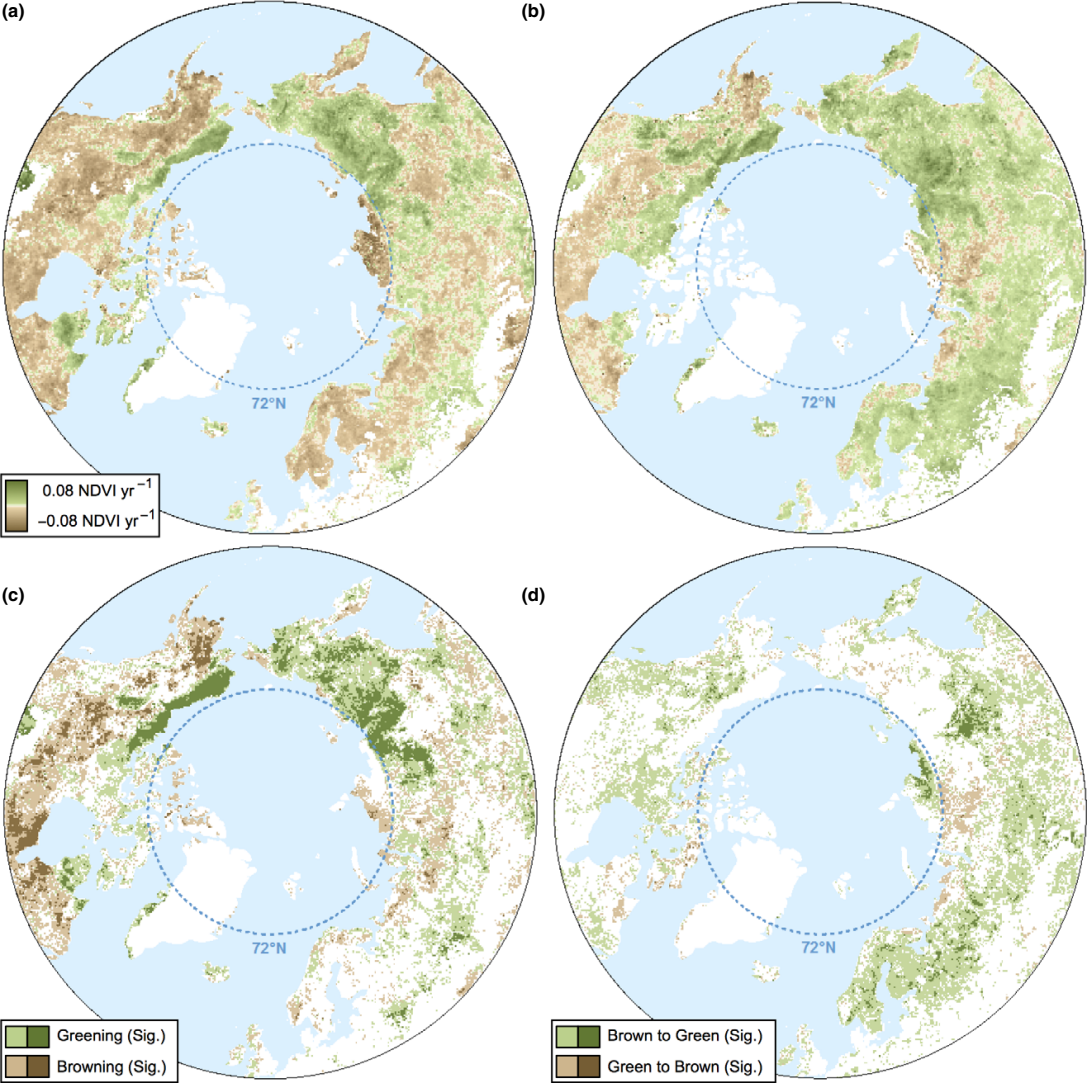
Trends in growing season NDVI (GS-NDVI) for (a) GIMMS_g_ and (b) GIMMS_3g_. Trends were calculated using the Theil–Sen approach over the period 1982–2008, which is the common record between both data sets. Croplands and water bodies were excluded. For maps depicting significant trends only, see Figure S4. The bottom two panels show areas where (c) both products agree in GS-NDVI trend direction and (d) where the products disagree. Areas where GIMMS_g_ indicated browning and GIMMS_3g_ indicated greening are labeled ‘Brown to Green’ and areas where GIMMS_g_ showed greening and GIMMS_3g_ showed browning are labeled ‘Green to Brown’. Dark colors indicate significant greening and significant browning (*P *<* *0.05).

Long-term trends in vegetation productivity (1982–2008), estimated using the GIMMS_g_ and GIMMS_3g_ data (Fig. 1a and b), agreed in 62% of the naturally vegetated high latitude region, excluding areas north of 72°N (Fig. 1c and Table 1). That is, 62% of grid cells in the study area showed a GS-NDVI trend estimated using the Theil–Sen approach, in the same direction, either greening or browning. In over half of this area (38.2%), the two data sets showed greening trends (i.e., GS-NDVI increased), and in slightly less than half (23.9%) they showed browning trends (i.e., GS-NDVI decreased). Areas where the newer GIMMS_3g_ data set confirmed the greening trends observed in the GIMMS_g_ data set were concentrated in the North American tundra (including the north slope of Alaska), which has exhibited some of the most consistent changes in GS-NDVI in recent decades, as well as in northeastern Siberia, particularly the northern reaches of the Lena river basin, where needle-leaf deciduous forests dominate. Conversely, GIMMS_g_ and GIMMS_3g_ both showed browning trends in the North American boreal forest, where GS-NDVI declines were relatively abundant (using the GIMMS_3g_ data, 60% of this area showed declines in GS-NDVI, and in 17% of the area these were statistically significant) (Fig. 1a and b).

Across the high northern latitudes, the GIMMS_3g_ data set indicated a markedly greater area with greening trends than the GIMMS_g_ data set did (Fig. 1d). The GIMMS_g_ and GIMMS_3g_ data sets showed opposite GS-NDVI trends in 38% of the study area, predominantly in areas where GIMMS_3g_ indicated greening trends while GIMMS_g_ indicated browning trends (34%). The reverse, i.e., areas that showed greening trends in GIMMS_g_ but browning trends in GIMMS_3g_, only occurred in a very small portion of the study area (<5%; Table 1), despite GIMMS_3g_ showing lower GS-NDVI trends than GIMMS_g_ in 25% of the area. Most of the revisions from browning to greening trends in GIMMS_3g_ occurred in the needle-leaf evergreen and mixed-leaf forests in European Russia, Scandinavia, interior Alaska, and southeastern Siberia, particularly the southern sections of the Lena river basin (Figure S5). The very small area that emerged as browning in GIMMS_3g_, but greening in GIMMS_g_ is located in central Siberia (Fig. 1d).

**Fig 2:**
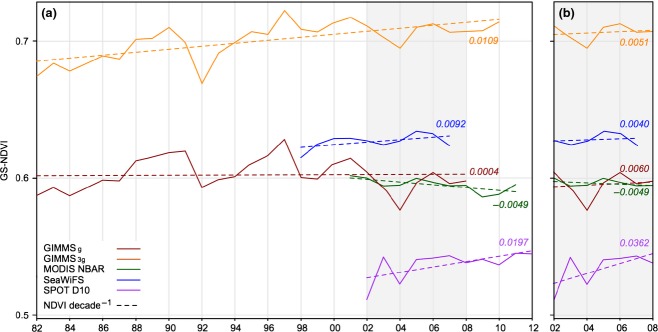
(a) Mean annual GS-NDVI for GIMMS_g_, GIMMS_3g_, MODIS NBAR, SeaWiFS, and SPOT D10 at naturally vegetated areas north of 50°N for the full available record for each product, and (b) the common record (2002–2008) of all data sets, excluding SeaWiFS which uses the period from 2002 to 2007.

**Fig 3:**
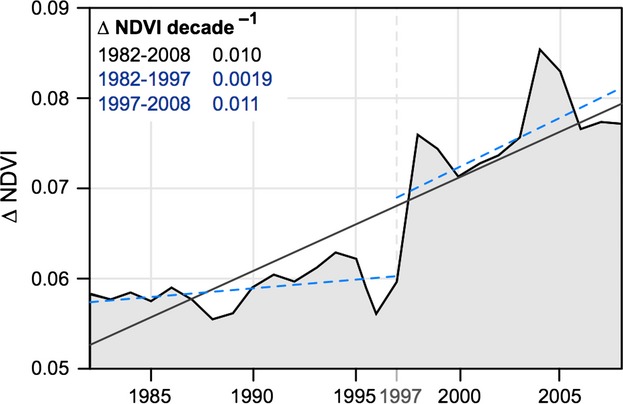
The annual difference between mean GS-NDVI in GIMMS_3g_ and GIMMS_g_ for naturally vegetated area between of 50°N and 72°N. The average rate of change (slope), estimated using least squares regression, is shown by the solid line. The dotted lines show the rate of change before and after 1997.

The tendency of GIMMS_3g_ to indicate greater and more widespread increases in vegetation productivity than its predecessor is evident at the pan-arctic scale (i.e., north of 50°N), where GIMMS_g_ and GIMMS_3g_ indicated an average trend of 0.0004 NDVI decade^−1^ and 0.0109 NDVI decade^−1^, respectively (Fig. 2a). The difference in mean GS-NDVI between the two products (Δ-NDVI) increased at an average rate of 0.010 NDVI units decade^−1^ (Fig. 3). This increase is concentrated, in a stepwise change, between 1997 and 1998, and has continued to diverge since then at a rate of 0.011 decade^−1^. In contrast, prior to 1997, divergence between the data sets is negligible at 0.0019 decade^−1^ (Fig. 3).

### GIMMS compared to MODIS, SeaWiFS, and SPOT

**Fig 4:**
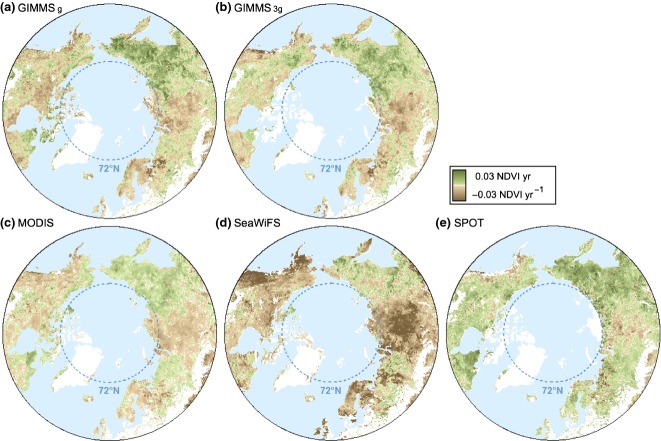
Trends in GS-NDVI derived from (a) GIMMS_g_, (b) GIMMS_3g_, (c) MODIS NBAR, (d) SeaWiFS, and (e) SPOT D10 using the common record (2002–2008*) and estimated using the Theil–Sen approach. Significant trends are shown in Figure S6. *SeaWiFS uses the period 2002–2007.

When averaged over the entire study area, from 2002 to 2008, the GS-NDVI trends for GIMMS_g_, GIMMS_3g_, SeaWiFS, and SPOT indicated greening while MODIS revealed browning. The greening trend seen in the SPOT data was considerably larger than that of the other data sets (0.0362 decade^−1^ vs. ≤0.006 decade^−1^, Fig. 2b). GIMMS_g_, GIMMS_3g_, and MODIS showed a similar abundance and spatial distribution of greening and browning trends, with greening concentrated in eastern Siberia and browning common in western Eurasia as well as much of boreal Canada (Fig. 4, Figure S6). However, across most of the study area, SeaWiFS and SPOT exhibited remarkably homogeneous browning and greening trends, respectively, in areas where GIMMS and MODIS showed strong greening and browning trends (dark colors in Fig. 4), and SeaWiFS and SPOT showed at least a small area with that same trend direction, albeit a much weaker trend.

**Fig 5:**
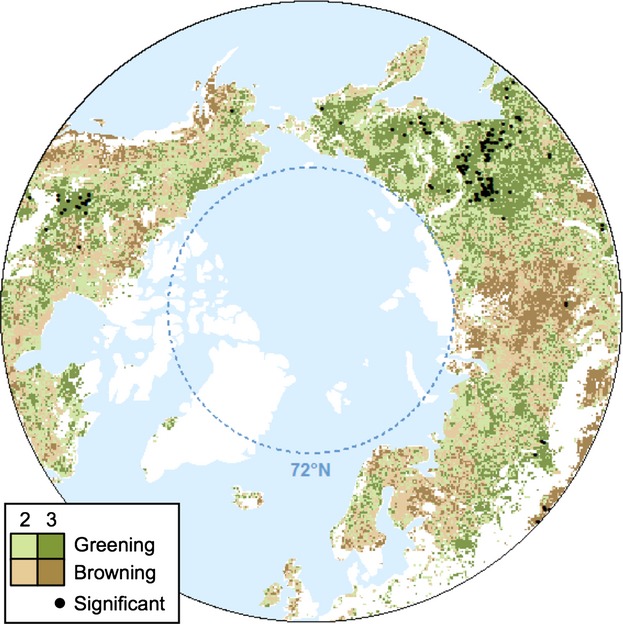
Agreement in the direction of GS-NDVI trends estimated for GIMMS_3g_, MODIS NBAR, and SPOT D10 over the common record (2002–2008). Dark colors show areas where GIMMS_3g_, MODIS NBAR, and SPOT D10 agree on the sign of the trend, while light colors indicate disagreement (i.e., two products agree and one product disagrees). Trends are assessed for statistical significance using the Mann-Kendall test (*P *<* *0.05). Black dots represent areas where all three data products indicate either significant greening or browning trends. Agricultural lands and areas north of 72°N are masked from the maps.

The agreement between GIMMS_3g_, MODIS, and SPOT was assessed over the common record on a per-pixel basis (SeaWiFS was not used because of its shortened record). The trend direction of the three products agreed in 46% of the study area, unanimously showing greening in 27.8% and browning in 12.2% (Table 2). Concordant greening, among these three products, was evident across most of eastern, and to a lesser extent western, Siberia as well as much of Canada; areas of concordant browning were mostly concentrated in central Siberia, and to a lesser extent, parts of North America including the western coast of Canada and southern Alaska (Fig. 5). The light brown and light green pixels in Fig. 5 indicate areas where two of the three aforementioned products showed a browning and greening trend, respectively, while the third product showed the opposite trend. Of this area, SPOT most often exhibited a greening trend where the other two products indicated a browning trend. Conversely, MODIS, and, to a lesser extent, GIMMS_3g_, most often indicated browning when the other two showed greening (Table 2). Black dots represent areas where all three NDVI products showed significant trends in the same direction (Fig. 5). While most of these are significant greening trends, located in eastern Siberia and western Canada, there are a few areas of agreement with significant browning trends.

**Table 2:** Percent of the study area where the GS-NDVI trend direction for GIMMS_3g_, MODIS NBAR, and SPOT D10 agree over the common record (first row) or where three of the aforementioned products agree and one disagrees (last three rows). In the later case, the one NDVI data set that disagrees with the other two is specified in the last three rows. Includes naturally vegetated land between 50°N and 72°N, excluding croplands

	Greening	Browning	Total
All agree	27.8	12.2	46.1
One disagrees			
GIMMS_3g_	3.8	12.9	16.7
MODIS NBAR	2.5	13.5	16.0
SPOT D10	18.5	2.7	21.2

**Fig 6:**
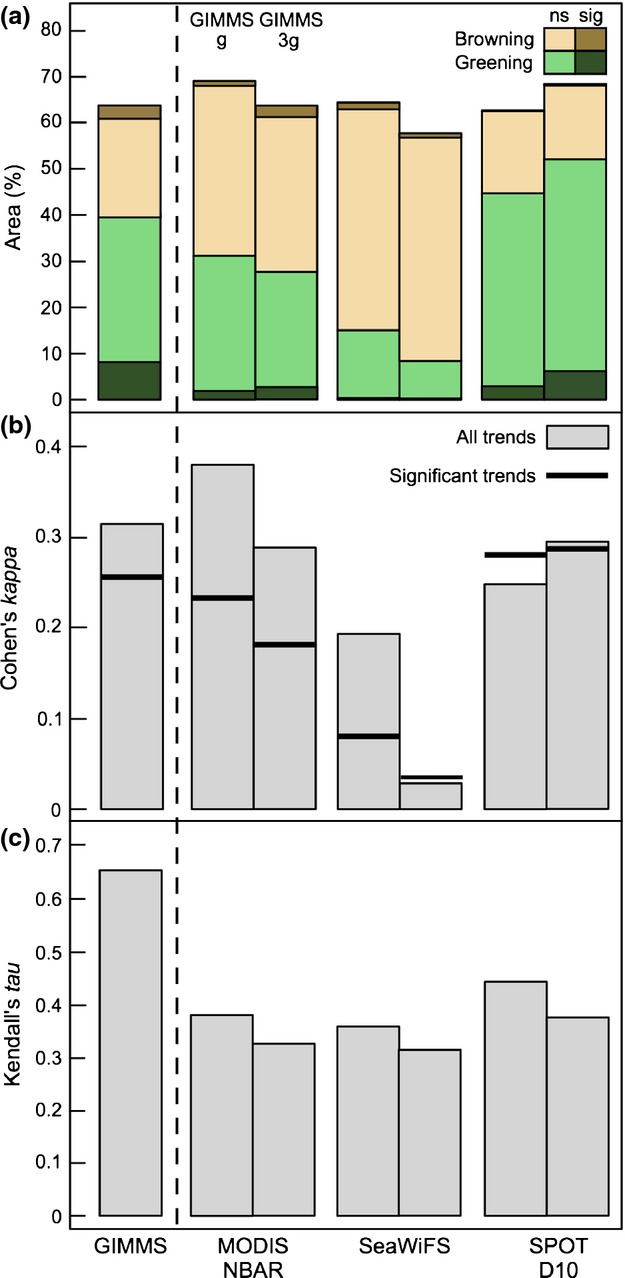
(a) The fraction of naturally vegetated area between 50°N and 72°N where GIMMS_g_ and GIMMS_3g_ (left and right bars, respectively) show similar trends in GS-NDVI as MODIS NBAR, SeaWiFS and SPOT D10 data products, over their respective periods of overlap (Figure S9). Green (bottom section of each bar) and brown (top section of each bar) indicate increases and decreases in GS-NDVI, respectively. Areas where both data sets show similar statistically significant trends are shown in dark colors. The Theil–Sen test was used to determine trend direction while the Mann–Kendall test was used to assess statistical significance. (b) Agreement between the sign of GS-NDVI trends in pairs of data products is estimated using Cohen's kappa. Bars indicate the level of agreement between all trends regardless of statistical significance and bold lines indicate agreement after non-significant trends are considered separately. (c) The correlation between product-pairs, based on per-pixel comparisons of detrended annual GS-NDVI values, quantified using Kendall's tau and averaged across all pixels. In a, b, and c, the bar to the left of the dotted line compares GIMMS_g_ to GIMMS_3g_. The bars to the right of the dotted line compare GIMMS_g_ and GIMMS_3g_ to MODIS, SeaWiFS, and SPOT.

Results in the following section are based on the maximum period of temporal overlap between the two products being compared (i.e., 2001–2010 when comparing GIMMS_3g_ and MODIS; Figure S7), the goal of this being to form more robust trends based on more than the 7 years of data common to the GIMMS, SPOT, and MODIS data sets. Comparing trends at a per-pixel scale revealed that GIMMS_g_ agrees in the most area with MODIS, while GIMMS_3g_ agrees best with SPOT. The area where each pair of products agrees is broken into greening and browning: in accordance with previous results, MODIS showed a similar amount of greening and browning with both GIMMS products, while GIMMS and SeaWiFS showed a disproportionate amount of browning in common and GIMMS and SPOT a disproportionate amount of greening in common (Fig. 6a).

Agreement can also be measured using Cohen's kappa, which takes into account agreement that occurs by chance (Cohen, 1960). Kappa was calculated on the GS-NDVI trends from the aforementioned pairs of products. Concurring with Fig. 6a, kappa values indicated that GIMMS_g_ agrees best with MODIS, while GIMMS_3g_ agrees the most with SPOT (Fig. 6b). SeaWiFS showed low agreement with both of the GIMMS products, owing to the greening trends in GIMMS that were ‘misclassified’ as browning trends in SeaWiFS. When calculated using significant trends, kappa revealed the same patterns, albeit at a lower level of agreement. Kendall's tau is a rank-based correlation coefficient that isolates year-to-year agreement from the longer term trends. Using this metric, GIMMS_g_ and GIMMS_3g_ showed relatively strong agreement (tau = 0.66), while GIMMS and the newer NDVI products showed less agreement (tau ≤ 0.45). When compared to MODIS, SeaWiFS and SPOT, GIMMS_g_ showed between 15% and 18% higher tau than that of GIMMS_3g_ (Fig. 6c). Of the newer data sets, GIMMS_g_ and GIMMS_3g_ ranked GS-NDVI most similarly with SPOT, followed by MODIS and SeaWiFS.

## Discussion

### Comparison of GIMMS_g_ and GIMMS_3g_

Our analysis revealed marked differences between the GIMMS_g_ and GIMMS_3g_ data sets, both in mean seasonal NDVI (Fig. 2), as well as long-term NDVI trends (Fig. 3). In general, greening was more prominent in GIMMS_3g_ than in the older GIMMS_g_ data set, particularly in the tundra, but also in needle-leaved evergreen and mixed-leaf forests (Figures S5 and S8). Greening of the tundra has been documented in some detail using GIMMS_g_ (Stow *et al*., 2004; Goetz *et al*., 2005; Verbyla, 2008; Forbes *et al*., 2010) and our study confirms these previous results using the revised GIMMS_3g_ data set. A number of previous studies using GIMMS_g_, and substantiated by comparisons with tree-ring measurements (Berner *et al*., 2011; Beck *et al*., 2013), reported browning across sizeable areas of boreal forest, particularity in the high-density needle-leaved evergreen forests of North America (Bunn & Goetz, 2006; Verbyla, 2008; Beck & Goetz, 2011). While we found that GIMMS_3g_ corroborated previous reports of browning in some regions (e.g., interior Alaska and central Canada) over similar timeframes, browning was generally less widespread in GIMMS_3g_ than it was in GIMMS_g._

The AVHRR sensors provide a unique record of earth surface measurements extending back three decades, yet generating a reliable time series from legacy sensors poses a significant challenge of considerable importance (see Tucker *et al*., 2005 for details). The GIMMS_3g_ data set is the latest iteration in this on-going effort and is an indispensable asset for the study of global ecosystem processes. As with any remote-sensing product, it remains an important task to validate these new data against *in situ* measurements of ecosystem processes. Efforts comparing GIMMS_3g_ NDVI with field measurements (including tree-ring width and maximum latewood density) have just started (Bunn *et al*., 2013), but further efforts are needed to link GIMMS_3g_ NDVI with ecosystem processes. In addition to comparing GIMMS with field measurements, further insight can be attained through cross-sensor comparisons.

### Cross-sensor comparison

The availability of NDVI data from different satellite platforms increasingly provides the opportunity to assess the robustness of any single data set (Brown *et al*., 2006; Yin *et al*., 2012). Over the common record, the GIMMS data sets most closely resembled MODIS with respect to spatial patterns in the direction of NDVI trends, each showing roughly half greening and half browning (Fig. 4). SPOT and SeaWiFS showed greening and browning trends, respectively, in three quarters of the study area, widely disagreeing with both GIMMS and MODIS. The widespread greening observed by SPOT could be attributed to documented differences in reflectance between the SPOT VGT1 and VGT2 sensors (Fensholt *et al*., 2009). While the ubiquitous browning trends in SeaWiFS are not documented, they may be related to sensor degradation that was inadequately accounted for in postprocessing. If the SeaWiFS series has degraded with time and the SPOT VGT series is inadequately corrected for factors that influence temporal consistency, then the focus of earth observation studies at high northern latitudes is currently heavily reliant on the most recent GIMMS_3g_ NDVI series and, importantly, the overlapping period of MODIS data. MODIS is particularly important, in that it represents the best-calibrated, highest quality data sets currently available, against which other multi-sensor times series can be compared. In that regard, the period of overlap between GIMMS_3g_ and MODIS suggests that the latter captures spatial and temporal patterns of NDVI reasonably well.

Through this study, we aimed to assess how GIMMS_3g_ compared to its predecessor GIMMS_g_, as well as to newer data sets from the more modern sensors: MODIS, SeaWiFS, and SPOT. Spatially, the agreement between GIMMS_3g_, MODIS, and SPOT is concentrated in central and eastern Siberia, central Canada and much of Alaska (specifically, the interior and the North Slope). In general, these are the same areas in which GIMMS_g_ and GIMMS_3g_ agree over the period from 1982 to 2008. Although our analysis is based on the BRDF-corrected MODIS and SPOT data sets (MOD43A4 and SPOT D10), their non-BRDF counterparts (MOD13A3 and SPOT S10) showed the same patterns with respect to distribution of greening and browning trends (Figure S10), interannual agreement (Figure S11) as well as agreement with GIMMS_g_ and GIMMS_3g_, although the agreement is greatest between GIMMS and the BRDF-corrected data sets (Figure S9).

In addition to assessing agreement based on GS-NDVI trends, a rank-based correlation coefficient, Kendall's tau, was used to test agreement between pairs of sensors with respect to year-to-year variability. We found that GIMMS_g_ NDVI consistently ranked years more similarly to that of MODIS, SeaWiFS, and SPOT than did GIMMS_3g_ (Fig. 6c). This is consistent with the higher agreement in GS-NDVI trends between GIMMS_g_ and MODIS and SeaWiFS (Fig. 6a and b). However, it is somewhat surprising that even at interannual time scales (with a considerably higher signal-to-noise ratio) the agreement among sensors is generally also limited. This points to a set of uncertainties in the satellite data sets that we analyzed, spanning both interannual (e.g., cloud contamination, effects of snow, etc.) and longer term (e.g., orbital drift, sensor degradation, etc.) time scales.

### Challenges in cross-sensor comparisons

Although cross-sensor comparisons are needed to assess the quality of any one NDVI data set, differences in sensor specifications, including resolution, spectral bandwidth and calibration, can make these comparisons difficult. Trends calculated using the relatively short period of overlap between GIMMS_g_, GIMMS_3g_, MODIS and SPOT are less reliable and result in fewer significant trends, while the 27-year common record between GIMMS_g_ and GIMMS_3g_ allowed for a more thorough comparison. Differences in spectral bandwidths among sensors can lead to NDVI responding to slightly different aspects of plant physiology and vegetation and landscape structure, which further complicate cross-sensor comparisons.

## Conclusions

We observed a large difference between the GIMMS_g_ and GIMMS_3g_ data sets, both in the magnitude of NDVI as well as in the direction of GS-NDVI trends, and therefore found it was necessary to provide a comprehensive comparison with the modern, higher quality NDVI data sets derived from the MODIS, SeaWiFS, and SPOT sensors. Our analysis revealed that with respect to GS-NDVI trends, both SPOT and SeaWiFS were biased towards greening and browning respectively. MODIS however, showed reasonably good similarity with GIMMS, both in the spatial distribution of trends and the individual NDVI measurements, agreeing in relatively equal areas of greening and browning. We found greening trends in Siberia and the North Slope of Alaska as well as browning trends in boreal North America to be robust among sensors, however in other regions, such as Scandinavia and central and western Siberia, GS-NDVI trends depend on the data set and thus need additional verification. Consistent with previous studies (Tucker *et al*., 2005; Beck *et al*., 2011a), we recommend that GIMMS be used in conjunction with MODIS and its successor, the Visible Infrared Imaging Radiometer Suite (VIIRS), due to the aforementioned similarities as well as the biases present in SPOT and SeaWiFS. Although MODIS showed more agreement with GIMMS_g_ than its successor, the increased spatial extent of GIMMS_3g_ (north of 72°N) alone makes it better suited for high latitude studies.

Because legacy AVHRR data are invaluable to the monitoring of earth's biosphere, continued efforts to process consistent time series data are critical. Further, these data should be cross-calibrated with more recent sensors including the new VIIRS instrument that succeeds MODIS, as well as with field measurements. While this study was limited to a relatively short period of temporal overlap between GIMMS and the newer NDVI products, a more thorough analysis could be done when longer time series data are available from AVHRR, MODIS, VIIRS, and SPOT. The differences we observed in the time series trends among data sets further emphasize the need for comparison and validation with field data, which implies robust spatial scaling approaches based on multi-scale imagery.

## Supporting Information

Fig S1 Timeline of the National Oceanic and Atmospheric Administration (NOAA) satellites, carrying the AVHRR sensors used in the GIMMS record. **Figure S2.** Spectral response curves for the red and near-infrared (NIR) bands of the AVHRR, MODIS, SeaWiFS, and SPOT VGT sensors. **Figure S3**. A map of lag-1 autocorrelation for the GIMMS3g GS-NDVI time series. **Figure S4.** Significant trends in GS-NDVI from 1982 to 2008 in the (a) GIMMSg and (b) GIMMS3g data sets for areas north of 50°N and excluding croplands. **Figure S5.** Land cover map using a combination of the Circumpolar Arctic Vegetation Map (CAVM) and the Global Land Cover 2000 (GLC2000) map. **Figure S6.** Significant and non-significant trends in GS-NDVI derived from (a) GIMMSg, (b) GIMMS3g, (c) MODIS NBAR, (d) SeaWiFS, and (e) SPOT D10 using the common record (2002–2008) and estimated using the Theil-Sen approach. **Figure S7.** Timeline showing the years covered by each satellite-derived NDVI product used in this study. **Figure S8.** The fraction of the study area displaying greening and browning trends in GS-NDVI for each vegetation type considered in this analysis. **Figure S9.** The agreement between different NDVI products (including NBAR and non-NBAR products) measured using the Mann-Kendall trend test, Cohen's kappa, and Kendall's tau. **Figure S10.** The fraction of naturally vegetated areas at high northern latitudes displaying greening and browning trends in GS-NDVI for both NBAR and non-NBAR data sets. **Figure S11**. Mean annual GS-NDVI for GIMMSg, GIMMS3g, MODIS NBAR, MODIS Terra, SeaWiFS, SPOT D10, and SPOT S10 in naturally vegetated areas north of 50°N.
