# Supplementary material for: Vegetation productivity patterns at high northern latitudes: a multi-sensor satellite data assessment
Source: Glob Chang Biol. 2014 Jul 21;20(10):3147–58. doi: 10.1111/gcb.12647 (PMC4312854; doi:10.1111/gcb.12647)
Supplement: Fig S1 — Timeline of the National Oceanic and Atmospheric Administration (NOAA) satellites, carrying the AVHRR sensors used in the GIMMS record. Figure S2. Spectral response curves for the red and near-infrared (NIR) bands of the AVHRR, MODIS, SeaWiFS, and SPOT VGT sensors. Figure S3. A map of lag-1 autocorrelation for the GIMMS3g GS-NDVI time series. Figure S4. Significant trends in GS-NDVI from 1982 to 2008 in the (a) GIMMSg and (b) GIMMS3g data sets for areas north of 50°N and excluding croplands. Figure S5. Land cover map using a combination of the Circumpolar Arctic Vegetation Map (CAVM) and the Global Land Cover 2000 (GLC2000) map. Figure S6. Significant and non-significant trends in GS-NDVI derived from (a) GIMMSg, (b) GIMMS3g, (c) MODIS NBAR, (d) SeaWiFS, and (e) SPOT D10 using the common record (2002–2008) and estimated using the Theil-Sen approach. Figure S7. Timeline showing the years covered by each satellite-derived NDVI product used in this study. Figure S8. The fraction of the study area displaying greening and browning trends in GS-NDVI for each vegetation type considered in this analysis. Figure S9. The agreement between different NDVI products (including NBAR and non-NBAR products) measured using the Mann-Kendall trend test, Cohen's kappa, and Kendall's tau. Figure S10. The fraction of naturally vegetated areas at high northern latitudes displaying greening and browning trends in GS-NDVI for both NBAR and non-NBAR data sets. Figure S11. Mean annual GS-NDVI for GIMMSg, GIMMS3g, MODIS NBAR, MODIS Terra, SeaWiFS, SPOT D10, and SPOT S10 in naturally vegetated areas north of 50°N. [file gcb0020-3147-sd1.pdf]

Global Change Biology (2014), doi: 10.1111/gcb.12647

Supporting information for:

Vegetation productivity patterns at high northern latitudes: a multi-sensor satellite data assessment

KEVIN C. GUAY[1], PIETER S. A. BECK[2], LOGAN T. BERNER[1,3], SCOTT J. GOETZ[1], ALESSANDRO BACCINI[1] and WOLFGANG BUERMANN[4]

[1]*The Woods Hole Research Center, 149 Woods Hole Road, Falmouth, MA 02540-1644, USA,* [2]*Forest Resources and Climate Unit, Institute for Environment and Sustainability (IES), Joint Research Centre (JRC), European Commission, Via Enrico Fermi 2749, Ispra, VA 21027, Italy,* [3]*Department of Forest Ecosystems and Society, Oregon State University, 321 Richardson Hall, Corvallis, OR 97331-5752, USA,* [4]*Institute for Climate and Atmospheric Science, School of Earth and Environment, University of Leeds, Leeds LS2 9JT, UK*

Correspondence: Pieter S. A. Beck, tel. +39 0332 78 3671,
fax +39 0332 78 5230, e-mail: pieter.beck@jrc.ec.europa.eu

Supporting Figures

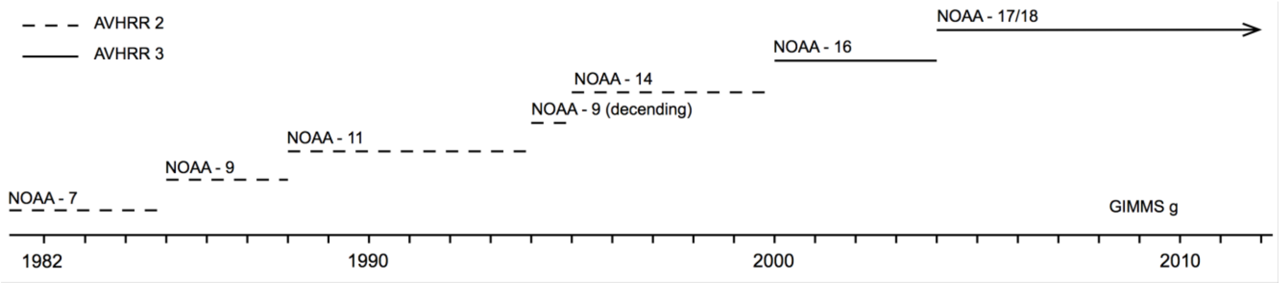

Figure S1 The series of NOAA satellites with an AVHRR sensor used in the GIMMS record. The second and third versions of the AVHRR (AVHRR/2 and AVHRR/3) sensor are shown using dotted and solid lines respectively.

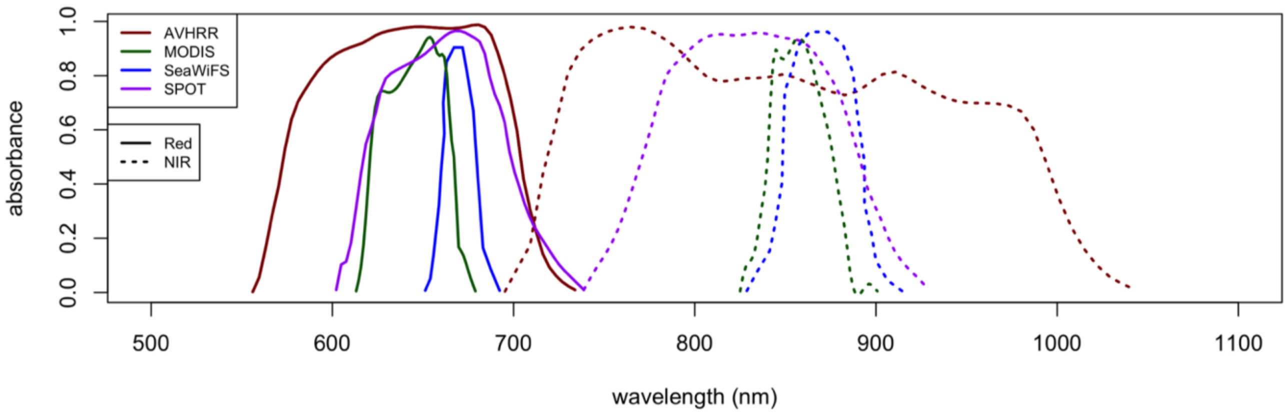

Figure S2 Spectral response curves for the red and near infrared (NIR) bands of the AVHRR, MODIS Terra, SeaWiFS and SPOT-VGT sensors.

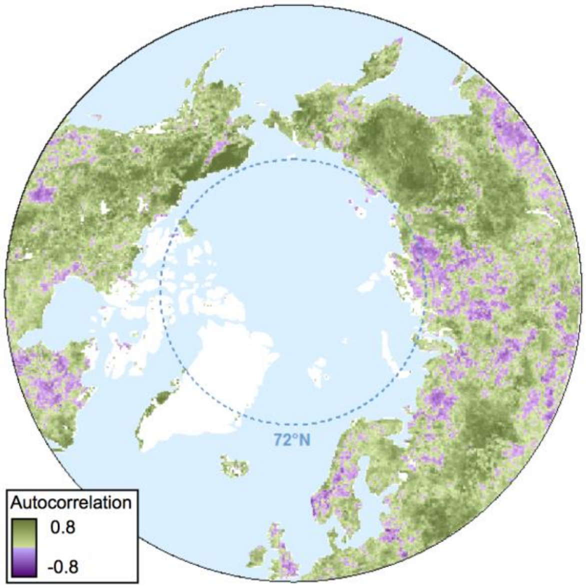

Figure S3 Autocorrelation calculated for the $GIMMS_{3g}$ GS-NDVI time series. The values are zero when no correlation exists and approach -1 (dark purple) or 1 (dark green) when a negative or positive lag-1 correlation exists, respectively.

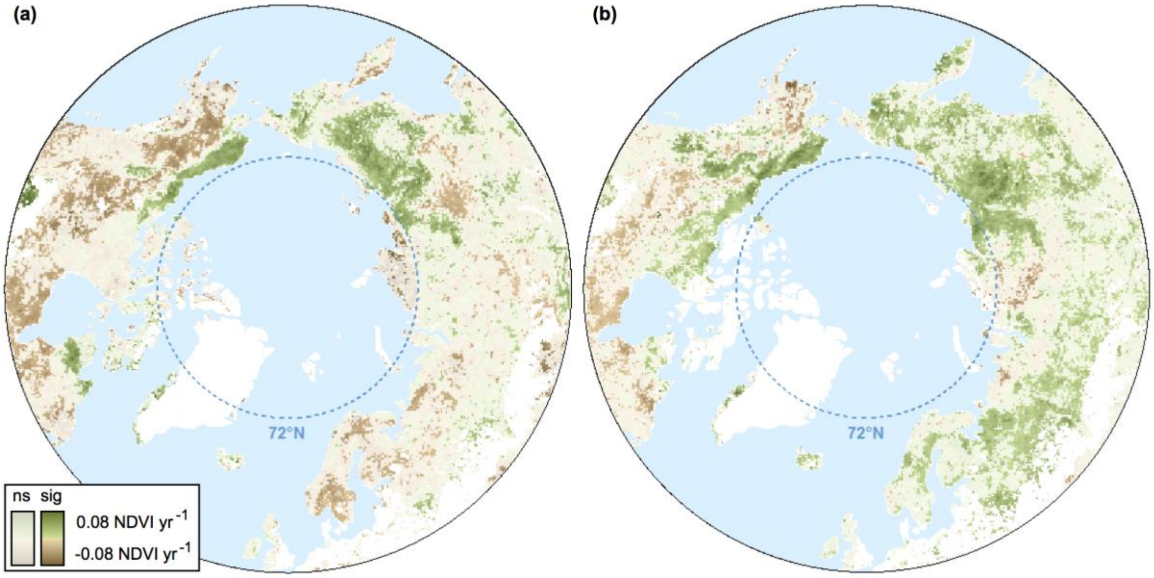

Figure S4 Significant trends in GS-NDVI from 1982 to 2008 in the (a) $GIMMS_g$ and (b) $GIMMS_{3g}$ data sets for areas north of 50°N and excluding croplands. Areas with non-significant trends ($p > 0.05$ following Mann-Kendall test) are shown in grey.

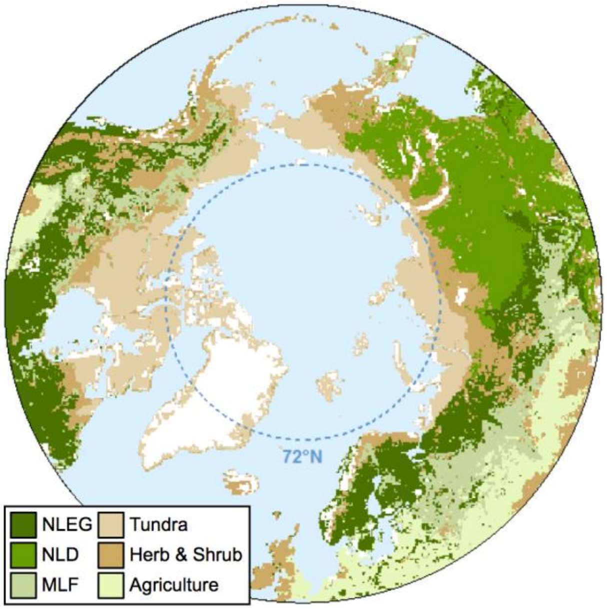

Figure S5 Land cover map relying on the Circumpolar Arctic Vegetation Map (CAVM) to distinguish the arctic tundra and on Global Land Cover 2000 (GLC2000) data to classify needle-leaf evergreen (NLEG), needle-leaf deciduous (NLD), mixed-leaf (MLF) forests as well as, Herb/ Shrub and Agriculture vegetation types. The agriculture class is not used in this study and is masked from the maps.

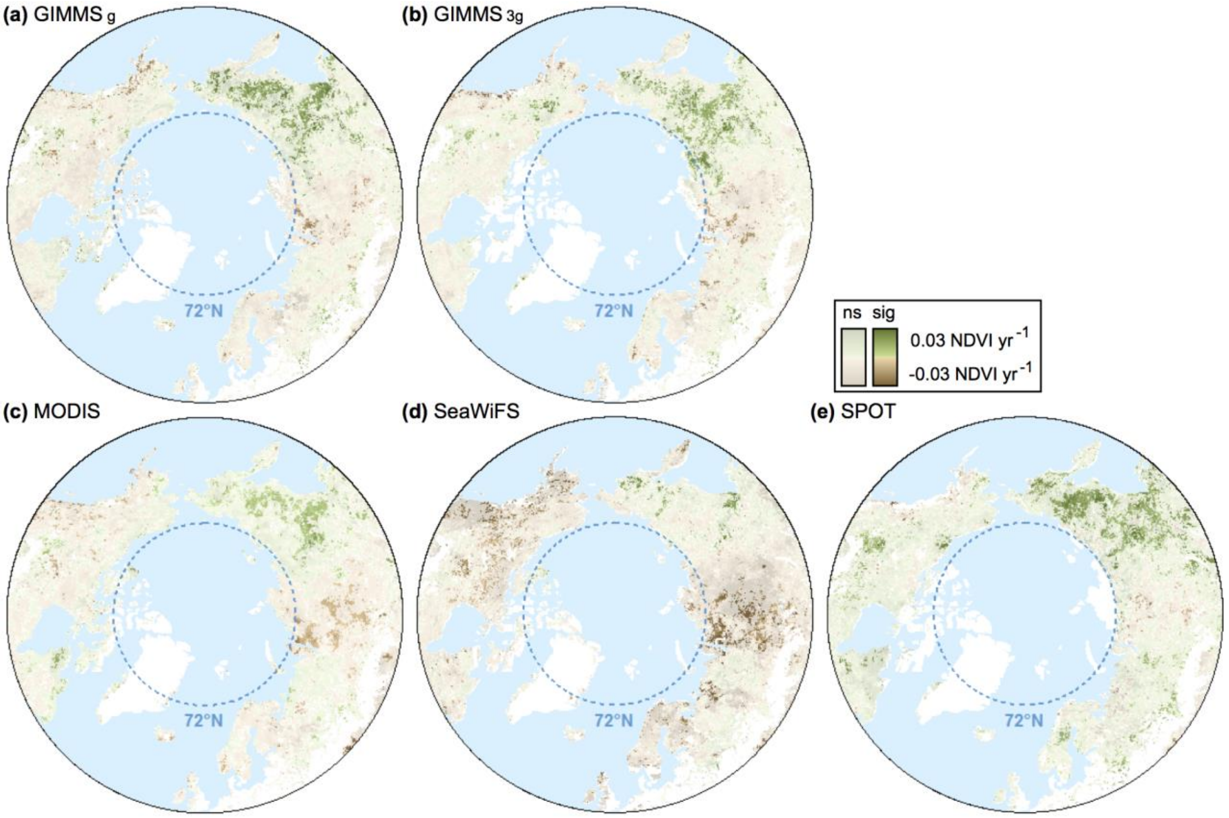

Figure S6 Significant and non-significant trends in GS-NDVI derived from (a) $GIMMS_g$, (b) $GIMMS_{3g}$, (c) MODIS NBAR, (d) SPOT D10 and (e) SeaWiFS using the common record (2002-2008) and estimated using the Theil-Sen approach. To eliminate scaling differences between NDVI-data products, trends are displayed in standard deviations (std. dev.) units, calculated from the trend slope values for each data product. Significant trends are shown in dark colors while non-significant trends are shown in light (faded) colors.

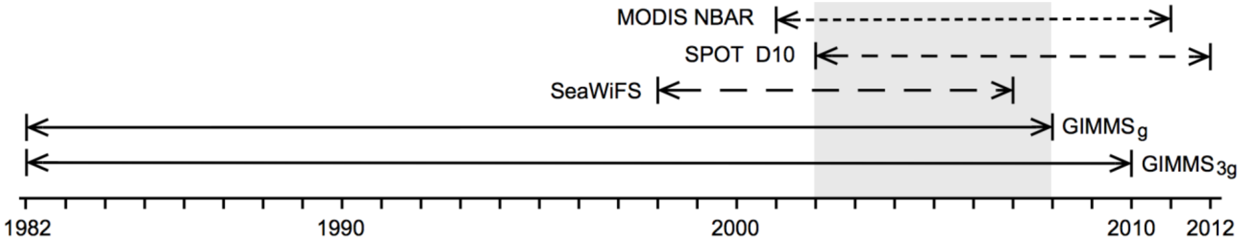

Figure S7 Timeline showing the years covered by each satellite-derived NDVI product. The last three years of the SeaWiFS record are not included because of satellite and sensor malfunctions. The common record (2002 – 2008) between $GIMMS_g$, $GIMMS_{3g}$, MODIS NBAR and SPOT D10 is shaded.

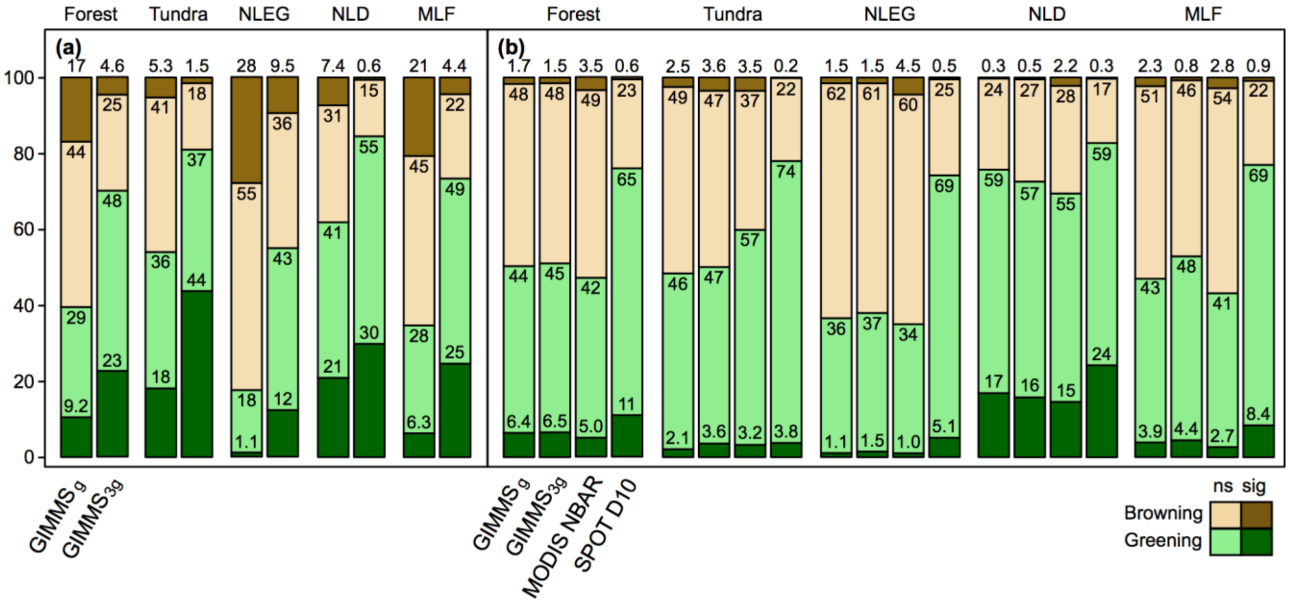

Figure S8 Percent of the study area that exhibited positive and negative GS-NDVI trends (i.e. greening and browning, respectively) using (a) $GIMMS_g$ and $GIMMS_{3g}$ over the period 1982-2008 and using (b) $GIMMS_g$, $GIMMS_{3g}$, MODIS NBAR and SPOT D10 data over the common record (2002-2008). Darker colors indicate statistically significant trends. From left to right, each section represents forest, tundra, needle-leaved evergreen (NLEG), needle-leaved deciduous (NLD) and mixed-leaf forest (MLF); the "Forest" class comprises NLEG, NLD, MLF, as well as herbaceous/shrubland (Figure S5).

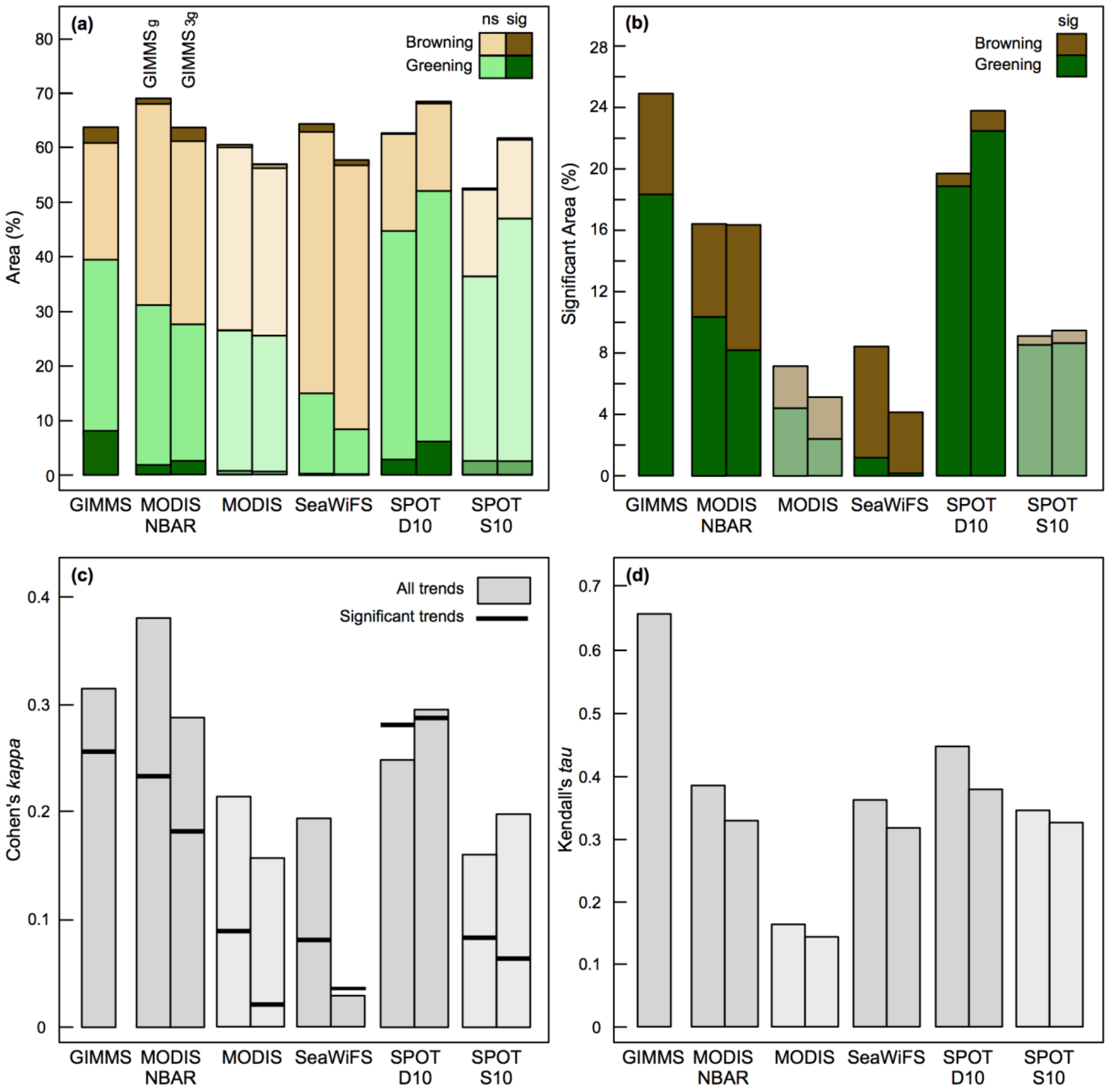

Figure S9 (a) The fraction of naturally vegetated areas at high northern latitudes where $GIMMS_g$ and $GIMMS_{3g}$ (left and right bars, respectively) show similar trends in GS-NDVI as MODIS NBAR, MODIS Terra, SeaWiFS, SPOT D10 and SPOT S10 data products, over their respective periods of overlap. Greening and browning denote increases and decreases in GS-NDVI, respectively, and areas where both data sets show similar statistically significant trends are shown in dark colors. The Theil-Sen test was used to determine trend direction while the Mann-Kendall test was used to assess statistical significance. (b) Similarity between statistically significant trends in GIMMS and other NDVI data products. Bars indicate areas where both data sets show similar statistically significant trends and are expressed as the fraction of the total area where either data set shows statistically significant trends. (c) Agreement between the sign of NDVI trends in pairs of data products estimated using Cohen's kappa. Bars indicate the level of agreement between all trends regardless of statistical significance and bold bar ends indicate agreement after non-significant trends are considered separately. (d) Correlation between product-pairs, based on per-pixel comparisons of detrended annual GS-NDVI values, quantified using Kendall's Tau and averaged across all pixels. Areas north of 72°N were excluded, because of lack of SPOT data. The MODIS Terra and SPOT S10 bars are transparent to isolate them from their BRDF-corrected counterparts (MODIS NBAR and SPOT D10).

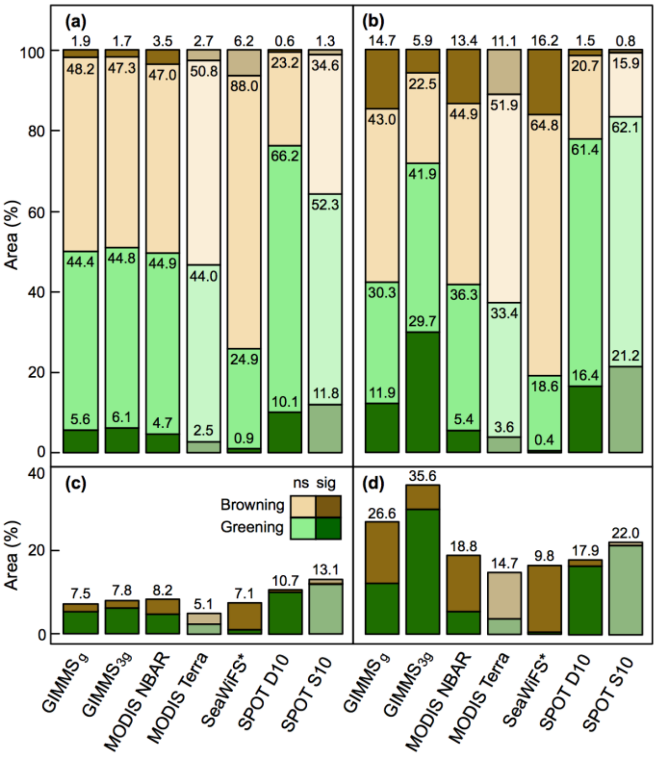

Figure S10 The fraction of naturally vegetated areas at high northern latitudes displaying greening and browning trends in GS-NDVI for both NBAR and non-NBAR datasets. (a) & (c) consider the common record (2002-2008) only, except for SeaWiFS (2002-2007). (b) & (d) consider the full record for each data product ($GIMMS_g$ 1982-2008; $GIMMS_{3g}$ 1982-2010; MODIS NBAR 2001-2011; MODIS Terra 2000-2012; SeaWiFS 1998-2007; SPOT D10 2002-2012; SPOT S10 1999-2012). (c) & (d) only show fractions where trends are deemed statistically significant based on the Mann-Kendall test ($p < 0.05$).

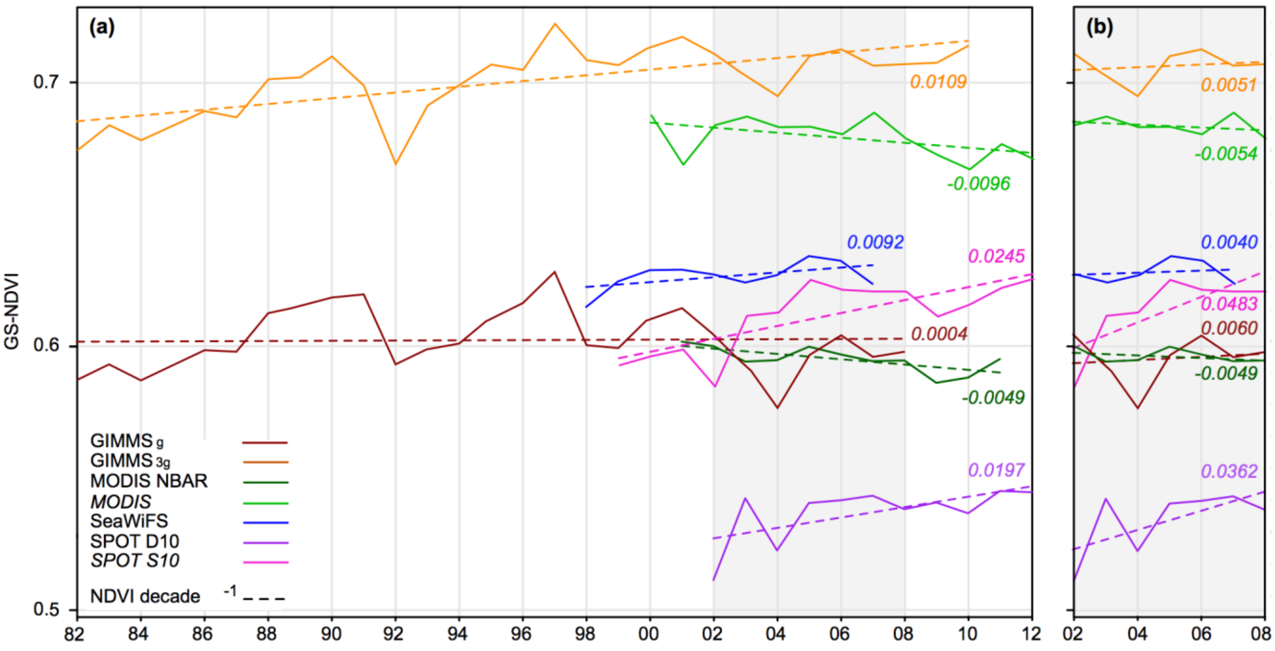

Figure S11 (a) Mean annual GS-NDVI for $GIMMS_g$, $GIMMS_{3g}$, MODIS NBAR, MODIS Terra, SeaWiFS, SPOT D10 and SPOT S10 in naturally vegetated areas north of 50°N for the full available record for each product, and (b) the common record (2002-2008) of all data sets, excluding SeaWiFS.

Supporting Methods

Spatial analysis

We compared the GS-NDVI data sets, over the naturally vegetated land in the high northern latitudes, north of 50°N. To divide this area into biomes and vegetation types, we used a combination of the Circumpolar Arctic Vegetation Map (CAVM; Walker *et al.*, 2005) and the Global Land Cover 2000 map (GLC2000), resampled to 24 km resolution using the dominant land cover type (Figure S5). The area classified here as Tundra includes all lands north of the tree line, i.e. the area mapped by the CAVM. Areas south of the latitudinal tree line that were classified as Herb and Shrub in the GLC2000 map generally represent areas of very sparse tree cover bordering the tree line, as well as alpine tundra and grasslands south of the boreal forest zone, and were only included here in the aggregate class "forest". The Mixed Leaf Forest (MLF) class, in this study, includes the mixed leaf type; broadleaved deciduous, open and closed (which at our scale of analysis usually occur in a mosaic with needle-leaf forest); burnt tree cover; tree cover and other natural vegetation (*sensu* GLC 2000).

We compared temporal productivity trends, depicted in different NDVI data sets, by mapping, on a per-pixel basis, increases and decreases in GS-NDVI, subsequently called greening and browning, respectively. This approach is robust to offsets or scale differences between NDVI data sets, which are to be expected because spectral response curves of red and near-infrared detectors differ between sensors (Figure S2). GS-NDVI trends were calculated over the full temporal record of each data set, as well as over the shared record of particular pairs of data set, and the temporal record common to all data sets (2002-2008). SeaWiFS, which contains severe anomalies starting in 2008, and is not standardized with regard to bidirectional reflectance, was not included in the common record. Analyses considering the $GIMMS_g$ data excluded areas north of 72°N, because of the discontinuity in the $GIMMS_g$ data set at that latitude.

Pre Whitening

It has been shown that significant temporal autocorrelation is evident in GIMMS GS-NDVI time series (Figure S3; Berner *et al.*, 2011; de Jong *et al.*, 2011). Trend analyses generally assume statistical independence between samples (i.e., years) and presence of autocorrelation in a time series violates this assumption, which can lead to an overestimate of effective sample size and of statistical significance (Zhang *et al.*, 2000). We therefore chose to use a nonparametric trend analysis technique that accounted for autocorrelation and which, in comparison to ordinary least squares (OLS) regression, was less sensitive both to non-normality of the distribution and to extreme values. We used an approach developed by Zhang *et al.* (2000), in a heavily cited article that investigated trends in temperature and precipitation across Canada over the 20th century. In developing their approach, the authors noted, "Using a limited number of series, we have compared the magnitude and statistical significance of the trend when the traditional linear model was fitted and when our approach was used. Generally, we have found that our approach produced a slightly smaller magnitude than that obtained by the traditional linear model, and, in some cases, trends identified as statistically significant using linear regression are not significant using our procedure due to positive autocorrelations in the time series (pg. 405)." This approach has been widely-used for analyzing NDVI time series (e.g. Ahmedou *et al.*, 2008; Mason *et al.*, 2008; Neeti *et al.*, 2011; Fensholt *et al.*, 2012; Berner *et al.*, 2013; Kim, 2013) and from our experience is more conservative than the OLS regression (e.g. Piao *et al.*, 2011; Epstein *et al.*, 2012), though appreciably less conservative than the Vogelsang test (e.g. Goetz *et al.*, 2005; Beck & Goetz, 2011).

Supporting Results

NDVI trends across vegetation types

Over the $GIMMS_g$ record (1982-2008), $GIMMS_{3g}$ data showed between 1.4 and 3.2 times more greening in forested areas north of 50°N than $GIMMS_g$ did, and up to 11 times more when only areas with statistically significantly greening were considered (Figure S8a). At the same time, the $GIMMS_{3g}$ data shows 1.8 to 2.5 times less browning, depending on the forest type, than the $GIMMS_g$ data did, and up to 12 times less when only areas with significant browning trends were considered. Mixed-leaf forest showed the largest shift from browning in $GIMMS_g$ to greening in $GIMMS_{3g}$, followed by needle-leaved evergreen and then needle-leaved deciduous forests. While the needle-leaved evergreen and mixed leaf forest (as well as the combined forest class), showed a larger area with significant browning than greening in the $GIMMS_g$ data set, in the $GIMMS_{3g}$ data set all vegetation types showed a greater area with greening trends than with browning, regardless of significance.

While the largest differences between long term trends in $GIMMS_g$ and $GIMMS_{3g}$ occur in forested areas, and particularly in the areas mapped as significantly browning, a similar pattern was observed to a lesser extent over the arctic tundra, where the $GIMMS_{3g}$ data indicated almost 1.5 times more greening than did the $GIMMS_g$ data, or 2.4 times more when only statistically significant greening was considered (Figure S8a).

Over the common record, SPOT-VGT exhibited the greatest area of greening across all vegetation types (up to 1.9 times more greening than the other NDVI products). Conversely, the MODIS data showed less greening in forested areas than SPOT and both GIMMS datasets, although relative to SPOT data, MODIS data showed similar amounts of greening and browning as both GIMMS datasets (Figure S8b). In the arctic tundra, however, MODIS showed 10% more of the study area greening than either GIMMS data set did, but still much less than SPOT data did.

Supporting References

Ahmedou O, Nagasawa R, Osman A, Hattori K (2008) Rainfall variability and vegetation dynamics in the Mauritanian Sahel. *Climate Research*, **38**, 75–81.

De Jong R, de Bruin S, de Wit A, Schaepman ME, Dent DL (2011) Analysis of monotonic greening and browning trends from global NDVI time-series. *Remote Sensing of Environment*, **115**, 692–702.

Epstein HE, Raynolds MK, Walker DA, Bhatt US, Tucker CJ, Pinzón JE (2012) Dynamics of aboveground phytomass of the circumpolar Arctic tundra during the past three decades. *Environmental Research Letters*, **7**, 015506.

Kim Y (2013) Drought and elevation effects on MODIS vegetation indices in northern Arizona ecosystems. *International Journal of Remote Sensing*, **34**, 4889–4899.

Mason JA, Swinehart JB, Lu H, Miao X, Cha P, Zhou Y (2008) Limited change in dune mobility in response to a large decrease in wind power in semi-arid northern China since the 1970s. *Geomorphology*, **102**, 351–363.

Neeti N, Rogan J, Christman Z et al. (2011) Mapping seasonal trends in vegetation using AVHRR-NDVI time series in the Yucatán Peninsula, Mexico. *Remote Sensing Letters*, **3**, 433–442.

Piao S, Wang X, Ciais P, Zhu B, Wang T, Liu J (2011) Changes in satellite-derived vegetation growth trend in temperate and boreal Eurasia from 1982 to 2006. *Global Change Biology*, **17**, 3228–3239.

Walker DA, Raynolds MK, Daniëls FJA et al. (2005) The Circumpolar Arctic vegetation map. *Journal of Vegetation Science*, **16**, 267.
